# Reports on the Hospitals of the United Kingdom

**Published:** 1909-10-16

**Authors:** Henry Burdett


### REPORTS
ON THE
### Hospitals of the United Kingdom.

By SIR HENRY BURDETT, K.C.B., K.C.V.O.

### III.—ROYAL HANTS COUNTY HOSPITAL, WINCHESTER.

This hospital was founded in 1736, when it stood on a site in a low-lying part of the town, with an open space at the back. It was rebuilt in 1868 on the straight pavilion plan, with a balcony at the end of each ward, and represents one of the best types of hospitals of its date. The wards are attractive and well kept, and present an inviting appearance to the visitor. It contains 108 beds, the average number occupied being about 100. The grounds at the back are pleasant and extensive, and the system of drainage has been carefully planned and is maintained in a thorough state of efficiency by constant inspection and flushing.

### The Hospital Spirit.

We have often said that the modern hospital, well administered and up to date, is a place which thoughtful members of the public should make it their business to visit and study. Here they will find an atmosphere of active, self-denying effort, where every member of the resident staff takes a pride in doing his best to keep the hospital, down to the minutest detail, in a high state of efficiency. In the course of our inspections, owing to the difficulty of getting from place to place, we have frequently arrived at a hospital in the late afternoon, as we did at Winchester; but whether our visit was early or late we have always found a welcome, and the utmost readiness to show everything as it was, so that we have had the advantage of seeing each hospital in its working state, and can so form a true estimate of the relative efficiency of every department. We take it that this feature of British hospital administration is one which makes the best of our hospitals the models for the most ambitious managers of every country to follow.

All that we have here written applies in an especial manner to the Royal Hants County Hospital at Winchester. There we found the best spirit animating all the resident staff, who devote themselves to their work and make it the first consideration of their lives. If the resident staff were alone responsible for the administration of this hospital, it could not fail to be as nearly perfect as it is possible to make an institution of this type. The voluntary system of hospital support and management has many merits, and has on the whole produced better results than that of any other system known to the modern world. Its grave defect is that circumstances may make it difficult to secure the services on the Board of Management of active men of business with large and generous views, who take a pride in their hospital and are unfailing in their efforts to keep it smart and well administered in every particular. Winchester is one of the most interesting cities in England, but we should imagine that the relatively few men of business amongst its citizens are unable for the most part to lend a hand in the administration of the affairs of this hospital. Were this not so, it is impossible to believe that certain easily removable defects would be permitted to continue to mar much which is so excellent and exemplary.

### Wanted, an Active Administrator.

The defects arise mainly from the enforcement of economy with so rigid a hand as to reduce it to a fault. Were this not so it would be impossible for members of the Committee to visit the hospital frequently and to tolerate the relatively dirty and unattractive appearance of the interior, to which attention has often been called, and which could be remedied at relatively small cost by redecoration in attractive colours by means of washable distemper. The same rigid economy imposes upon the resident staff, or some of them, a burden of excessive duty, which is unjust to themselves and inimical to the proper execution of the necessary work. Seeing that the hospital has a private nursing staff, and that the training of nurses is systematically carried on to the advantage of the institution, it is surprising to find that the Committee has not spontaneously provided Miss Carpenter, the most efficient matron and lady superintendent, with a competent assistant when the need for the appointment of such an officer is so real and apparent. Again, having regard to the active surgical and medical work carried on in the institution at the present time, it would seem to be desirable that the policy adopted by other similar hospitals should be followed, and that an extra medical resident should be appointed.

We might indicate other directions in which the wise expenditure of a little money, and the strengthening of the staff would tend to do justice to those responsible for the working of the institution and to the Committee in the management of its affairs. No money is better spent than by the liberal apportionment of salaries to competent officials, and we hope that the Winchester Committee will consider favourably the suggestions we have ventured to make, and that it will proceed without delay to set its house in order. We take it that members of the Committee must be justly proud of the results of the devotion of the resident staff at this hospital, and, if they are, and they possess that interest in the hospital which we have a right to attribute to them, they will not delay to remedy the existing defects which claim their immediate consideration.

### Financial Considerations.

Realising the economical timidity of the Committee, as exhibited by the state of affairs we have just described, it may be useful to point out that the time has come when the example set by other county hospitals might profitably be followed with a view to increase the annual revenue of this hospital. The number of patients whom annual subscribers are entitled to recommend is based upon a scale which is not just to the hospital. This scale is neither business nor charity. The average cost of each in-patient is stated in the report to have been during the last financial year £5 6s. 3d., and of each out-patient 3s. 9d. It is clear, therefore, that the scale of privileges which gives every subscriber of two guineas one in-patient and three out-patient tickets, representing, if they are used, a cost to the hospital of £5 17s. 6d., is one which calls for immediate revision. We have always been in favour of the abolition of tickets, but we recognise that in the case of county hospitals custom often makes it undesirable to abolish tickets altogether, though we have never found any residents in an English county who fail to respond and support every hospital committee, the members of which, as men of business, revise the scale of privileges for subscribers, and place it upon a more business-like and fair basis. Wherever such revision has taken place the revenue of that county hospital has increased considerably, and its committee have been able to do more for the patients directly by increasing the efficiency of the institution, and indirectly for the subscribers by making the county hospital fulfil the purpose for which it was established to a much greater extent than it was possible for them to do under the old scale of privileges. It is surely time, therefore, for the members of the Committee of the Royal Hants County Hospital to take up this question and to place it once and for all on a fair and business-like footing. If they will do this they will, we are confident, find that the increased revenue will enable them to do many things which might and ought properly to be done. As matters stand at present, were it not for the considerable income derived from the earnings of the private nurses, much of which ought by rights to revert to the nurses who earn the money, there would be a deficiency shown in the accounts for the year. In any case, the available income, as matters stand at present, is amply sufficient to justify the Committee in making the additions to the staff we have suggested, and at the same time give instructions for the redecoration of the interior of the hospital, or those portions of it which at the present time are dirty and unattractive.

We hope that the days of starvation in little things at this hospital may soon pass away, and that the Committee, gaining some of the spirit which animates its resident staff, may vie with the latter in doing its utmost for the efficiency of the hospital in all departments. It is, by the way, a little remarkable that the name of the hospital should not be prominently displayed on the buildings. To the stranger it is not too easy to find, and the suppression of its name makes the difficulty greater. Why this reticence?

The New Nurses' Home.

The plan and arrangement of this home, and everything connected with it, reflect the greatest credit upon Miss Carpenter and those who are responsible for the plans. It is a pleasure to go over a nurses' home where every detail has been thought out in a practical manner before the plans were accepted, and where an earnest attempt has been made so to arrange every portion of the buildings that they may provide the maximum of comfort to the inmates and the minimum of cost to the management for their maintenance. These results we consider to have been successfully attained in the new nurses' home at Winchester. We hope to publish later a full description and plans of this home, for the buildings contain some novelties and many attractive details which give them a special interest for all who have to plan or provide buildings of this type.

**The Cardiff Infirmary, Newport and Monmouthshire, and Royal Hamadryad Seamen's Hospital, Cardiff,** will be reported on next week.

